# Imaging the cerebellum in post-traumatic stress and anxiety disorders: a mini-review

**DOI:** 10.3389/fnsys.2023.1197350

**Published:** 2023-08-14

**Authors:** Patricia Gil-Paterna, Tomas Furmark

**Affiliations:** Department of Psychology, Uppsala University, Uppsala, Sweden

**Keywords:** cerebellum, vermis, anxiety, stress, PTSD, human neuroimaging, MRI

## Abstract

Post-traumatic stress disorder (PTSD) and anxiety disorders are among the most prevalent psychiatric conditions worldwide sharing many clinical manifestations and, most likely, neural mechanisms as suggested by neuroimaging research. While the so-called fear circuitry and traditional limbic structures of the brain, particularly the amygdala, have been extensively studied in sufferers of these disorders, the cerebellum has been relatively underexplored. The aim of this paper was to present a mini-review of functional (task-activity or resting-state connectivity) and structural (gray matter volume) results on the cerebellum as reported in magnetic resonance imaging studies of patients with PTSD or anxiety disorders (49 selected studies in 1,494 patients). While mixed results were noted overall, e.g., regarding the direction of effects and anatomical localization, cerebellar structures like the vermis seem to be highly involved. Still, the neurofunctional and structural alterations reported for the cerebellum in excessive anxiety and trauma are complex, and in need of further evaluation.

## Introduction

The cerebellum is a highly organized brain region located in the posterior fossa, and most known for its role in motor coordination, and bodily posture and balance (reviewed in Caulfield and Servatius, 2013; Kandel et al., 2013). While accounting for only 10% of the total brain volume, the cerebellum harbors more neurons than the rest of the brain (Azevedo et al., 2009). By its anteroposterior direction, the cerebellum is divided in two hemispheres and its midline area, the vermis, forming in tandem the three main lobes: flocculonodular, anterior and posterior lobes, subdivided in lobules I-X that constitute the distinctive *folia* (Apps and Hawkes, 2009; Schutter, 2020). The cerebellar cortex congregates gray matter in its outer part, while white matter is found in the innermost part, innervating the three deep cerebellar nuclei: dentate, fastigial, and interposed nuclei (Kandel et al., 2013).

Beyond motor-related functions, cumulative evidence support that the cerebellum modulates higher order and executive functions, including prediction and error-based learning (Butcher et al., 2017; Sokolov et al., 2017; Uehara et al., 2018), associative and implicit learning (Timmann et al., 2010), episodic (Andreasen et al., 1999; Habas et al., 2009; Almeida et al., 2023) and working (Fafrowicz et al., 2023) memory systems, language (Murdoch, 2010; Mariën and Borgatti, 2018; King et al., 2023), as well as emotion regulation (Baldaçara et al., 2008; Lange et al., 2015; Caria and Grecucci, 2023). Lesion studies of the posterior cerebellum led to the description of the *cerebellar cognitive affective syndrome* (Schmahmann and Sherman, 1998) with a suggested cerebellar regulatory role in emotion and cognition through reciprocal connections with limbic and prefrontal regions.

Fear is a biologically basic emotion that has attracted considerable research interest due to its relevance for many clinical disorders, and the cerebellum is interconnected to brain regions comprising the fear circuitry (Apps and Strata, 2015). Animal and human studies have shown that the cerebellum is involved in fear conditioning and fear memories (Sacchetti et al., 2005; Timmann et al., 2010; Lange et al., 2015; Strata, 2015; Adamaszek et al., 2017; Frontera et al., 2020), which could be tied to the etiology of anxiety, trauma and stress-related disorders. Anxiety disorders are characterized by excessive fear and avoidance in presence of stimuli perceived as threatful, as well as heightened anticipation of threatening future events. Several clinical features are shared with post-traumatic stress disorder (PTSD) including fear and avoidance, hyperarousal, increased autonomic response, psychosomatic symptoms and trauma-related aversive memories. PTSD, which was separated from anxiety disorders in the fifth edition of the Diagnostic and Statistical Manual for Mental Disorders (American Psychiatric Association, 2013), is further characterized by hypervigilance and difficulties in maintaining concentration (Peters et al., 2021), and by difficulty in discriminating safety from threat cues (Williamson et al., 2021). The global lifetime prevalence rates have been estimated to 28.8% for anxiety disorders and 3.9% for PTSD (Kessler et al., 2005; Koenen et al., 2017). Comorbidity with other disorders, such as depression, is common (Whiteford et al., 2013).

The cerebellum has been largely understudied in comparison to traditional limbic regions like the amygdala, but recent imaging research findings indicate that the cerebellum is involved in the pathophysiology of anxiety disorders including social anxiety disorder (SAD), generalized anxiety disorder (GAD), panic disorder (PD), specific phobia (SP), as well as PTSD. Anxiety patients have been reported to display both altered cerebellar activity and connectivity with corticolimbic areas, and changes have been found after pharmacological interventions with antidepressants (e.g., Chin and Augustine, 2023) and psychological interventions such as cognitive behavioral therapy (e.g., Kindred et al., 2022). There is evidence of hyperactivity both in the cerebellum and amygdala in SAD patients (Tillfors et al., 2002; Evans et al., 2008), higher cerebellar baseline activity in PD (Sakai et al., 2005) and increased cerebellar activity in PTSD (Wang et al., 2016), although mixed results are found across disorders.

While it can be hypothesized that individual differences in cerebellar activation underlie reactivity to stressors (Moreno-Rius, 2019) and the risk for developing anxiety and stress-related disorders (Caulfield and Servatius, 2013), a clear understanding of how the cerebellum contributes to excessive anxiety and stress is lacking. The aim of this mini-review was to describe the main findings, at the cerebellar level, of human neuroimaging studies using structural (sMRI) or functional (fMRI) magnetic resonance imaging, in adult patients suffering from PTSD or anxiety (SAD, GAD, PD, SP) disorders.

## Methodology

**FIGURE 1:**
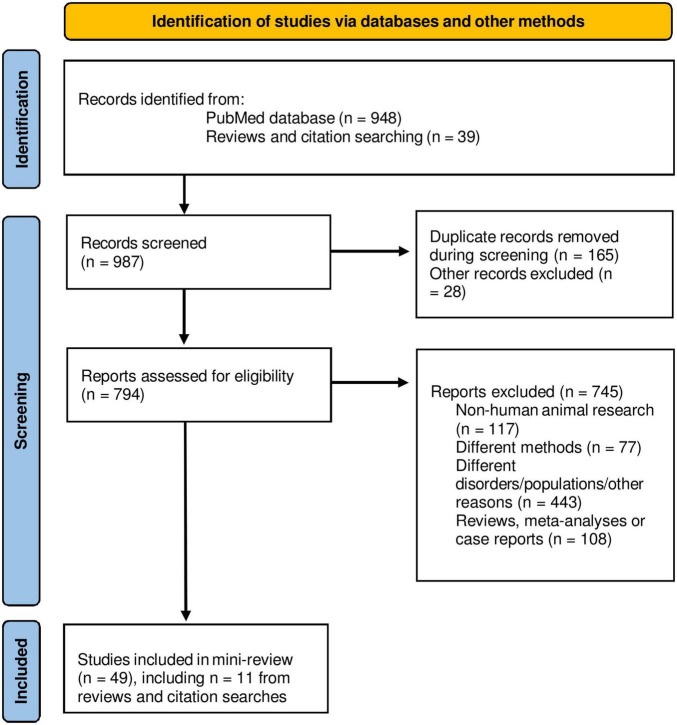
PRISMA flow diagram of the mini-review. Adapted from Page et al. (2021).

Only original MRI research papers published in peer-reviewed English-language journals reporting findings in the cerebellum were considered. An advanced electronic literature search in PubMed database^1^ without time restriction was carried out by using the following terms with the Boolean operator AND: “((cerebellum) AND (anxiety)) AND (MRI)” (290 results); “[(cerebellum) AND (stress)] AND (MRI)” (284 results). Additionally, advanced sub-searchings were performed for each of the target disorders: “cerebellum AND acute stress disorder” (*n* = 34); “cerebellum AND PTSD” (*n* = 94); “cerebellum AND generalized anxiety disorder” (*n* = 135); “cerebellum AND social anxiety disorder” (*n* = 69); “cerebellum AND panic disorder” (*n* = 24); “cerebellum AND specific phobia” (*n* = 18). Furthermore, recent review papers and citations were scanned for non-detected original trials, and 11 studies were added from 39 additionally scanned research articles. Figure 1 shows a flow diagram of the screening process. Research studies using sMRI or fMRI, with adult participants (>18 years of age) that had received a clinical diagnosis, in either patient-control or pre-post-treatment comparisons were included. Exclusion criteria were research articles that used: (I) another neuroimaging modality than sMRI or fMRI; (II) pediatric or adolescent populations, or healthy individuals only; (III) neuropsychiatric disorders different than the targeted PTSD/anxiety disorders; and (IV) meta-analyses, reviews or case reports.

## Results

From a total of 987 papers screened, 49 papers matching inclusion/exclusion criteria were selected: PTSD (*n* = 30), PTSD + SAD (*n* = 1), SAD (*n* = 9), GAD (*n* = 2), PD (*n* = 4), SP (*n* = 3). The total number of patients was 1,494 (600 males/894 females). A descriptive summary and imaging results are presented in Supplementary Table 1 (task-based fMRI studies) and Supplementary Table 2 (resting state rs-fMRI and/or sMRI studies) in Supplementary material. Task-based fMRI studies mainly used disorder-relevant challenges to obtain the neural activation maps during task (disorder-relevant stimuli) compared to control conditions (neutral stimuli). Most rs-fMRI studies measured functional connectivity (FC) between the cerebellum and other brain regions (intrinsic connectivity distribution), or at intra-cerebellar network level (intra-network FC). Structural MRI studies mostly used voxel-based-morphometry (VBM) to identify variations in gray matter volume (GMV). The vast majority of studies were cross-sectional comparing patients vs. controls while some were treatment studies evaluating pharmacotherapy and/or psychological interventions with pre-post within-group evaluation or treatment vs. placebo comparisons in patients. Two studies also used a machine learning approach to classify groups.

### Task-based fMRI studies

#### PTSD

The majority of fMRI studies on PTSD used trauma or autobiographical memory-related visual or mental imagery tasks or pain-related stimuli (see Supplementary Table 1). Mental imagery or script-driven imagery tasks are commonly used to evoke traumatic emotional states upon presentation of autobiographical written scripts of a traumatic event previously described by the participant, who is instructed to recall the event and think about it in the most lucid possible way (Douglas et al., 2019). Results are mixed, although task-related cerebellar hyperactivity is commonly reported, e.g., in the culmen and vermis (Ke et al., 2015), crus I and II (Awasthi et al., 2020), lobule VI (Rabellino et al., 2016; Naegeli et al., 2018), and lobule V (Terpou et al., 2019), whilst increased deactivation to reward stimuli was reported in right cerebellar crus II, and lobules VIIb and VIII (Elman et al., 2018). The vermis and left cerebellum that showed hyperactivation at baseline in PTSD, tended to decrease in a 2-year follow-up after the trauma (Ke et al., 2016). Lateral and left cerebellum also showed alteration in PTSD patients (Strigo et al., 2010; Vidotto et al., 2014; Chiasson et al., 2021). As glucocorticoids modulate stress and memory processes, such as emotional memory consolidation and recall, hydrocortisone is a potential medication for PTSD and fear-related disorders (e.g., Soravia et al., 2006; Grillon et al., 2011; Cawley et al., 2022). Following hydrocortisone administration in PTSD, Metz et al. (2019) found an increase in the left cerebellum during an autobiographical memory recall task, relative to a placebo group, whereas Douglas et al. (2019) reported a decreased blood flow in left cerebellum during script-driven trauma imagery in the hydrocortisone group.

#### Anxiety disorders

A variety of affective and disorder-relevant experimental tasks have been used in fMRI trials of anxiety disorders with a mixed pattern of results (Caseras et al., 2010; Nakao et al., 2011; Giménez et al., 2012; Petrowski et al., 2014; Schwab et al., 2020; Korgaonkar et al., 2021). One study in SAD reported increased activation in the bilateral cerebellum and vermis, during a social scrutiny exposure task, but activation in the left cerebellum was also reported in controls (Giménez et al., 2012). In this task, red and green dots were intercalated in the fMRI computer screen, and participants were told that during presentation of red dots, their facial expressions and postural movements would be close-up recorded (Giménez et al., 2012). Similarly, Nakao et al. (2011) found that both SAD and controls showed cerebellar activation during a social situation task, although controls displayed greater activation in left cerebellum. In PD, relatively decreased activation of the right cerebellum was reported during processing of emotional faces (Korgaonkar et al., 2021). In line with this, another study using a similar task noted decreased activation of the left cerebellum in PD patients with comorbid agoraphobia, relative to controls (Petrowski et al., 2014). A negative amygdala to cerebellum connectivity was found in GAD participants during implicit verbal memory tasks (Park et al., 2022), and in SAD patients undergoing cognitive-behavioral treatment positive changes in amygdala-cerebellar connectivity predicted less improvement (Sandman et al., 2020). Finally, fMRI studies on SP reported an increased bilateral activation of the cerebellum of participants with spider phobia and left cerebellum hyperactivation in blood-injection-injury phobia (Caseras et al., 2010) during processing of phobia-related visual stimuli. Schwab et al. (2020) found hyperactivation of the left cerebellum in spider phobic group after hydrocortisone administration targeting glucocorticoids-modulated aversive memories.

### Resting-state fMRI studies

#### PTSD

As listed in Supplementary Table 2, Rabellino et al. (2018) reported increased FC, between the left cerebellar IV–V lobes and the right fusiform gyrus and hippocampus, and also between the right IV–V cerebellar lobes with right posterior insula and planum polare in PTSD. In contrast, the PTSD group, as compared to controls, showed decreased FC in left Crus I to frontal gyrus. The same research group recently found that the FC between the right flocculus and the right hippocampus was increased in a PTSD dissociative subgroup compared to PTSD (Rabellino et al., 2022). Holmes et al. (2018) noted hyperconnectivity between the cerebellum and the supramarginal gyrus in PTSD, compared to controls. Nicholson et al. (2015) reported that the PTSD dissociative subgroup had increased FC between the basolateral amygdala and left culmen, and the same group (2020) found the crus I as a network region in the central executive network (CEN) classifying patient groups. Another five studies found decreased amplitude of low-frequency fluctuations in the right posterior cerebellum (Yin et al., 2011), decreased FC between the CEN and a cerebellar region (Vuper et al., 2021), dorsal anterior cingulate cortex and the cerebellum (Chen et al., 2019), right cerebellar vermis relative to the periaqueductal gray, bilateral culmen and left cerebellar lingual (Thome et al., 2017), and decreased FC between the cerebellum, dorsolateral and medial prefrontal cortices (Holmes et al., 2018). In another study, the amplitude of low-frequency fluctuations was increased in the right cerebellum of PTSD individuals (Bing et al., 2013). In studies using a support vector machine learning approach, the bilateral cerebellum was one the most informative regions separating patients with PTSD from controls at rest (Zhang et al., 2016), or remitted vs. persistent PTSD patient groups, measuring intra-network FC in Crus I, following a 12-week treatment with paroxetine (Yuan M. et al., 2018).

#### Anxiety disorders

Social anxiety disorder subjects, showed reduced resting state FC across different cerebellar subregions, especially in left Crus I with frontal areas (Yuan et al., 2017). Moreover, increased pretreatment FC in vermis Crus I relative to angular gyrus and right dorsolateral prefrontal cortex, predicted treatment response and symptom improvement. Another two studies of SAD found decreased FC in bilateral posterior cerebellum with bilateral putamen and right thalamus (Zhang et al., 2022), and decreased connectivity among left precuneus and left posterior cerebellum (Yuan C. et al., 2018). GAD was also marked by a reduced FC between right amygdala and cerebellum (Du et al., 2021), whereas in PD a decreased intra-cerebellar FC was found in right lobules V–VI, vermis, and left lobule VI of the cerebellum network (Ni et al., 2021).

### sMRI studies of cerebellar gray matter volume

#### PTSD

Increased cerebellum GMV has been noted in cerebellar lobules VIIb, VIIIa, and VIIIb of PTSD subjects (Sussman et al., 2016). Increased gray matter density in left cerebellum, but decreased in the frontal lobe, right amygdala and hippocampus, was reported in PTSD, compared to controls (Sui et al., 2010). Baldaçara et al. (2011, 2012) showed that PTSD participants had lower left cerebellar hemisphere and vermis volume, compared to resilient controls, and volume correlated negatively with PTSD symptomatology. Holmes et al. (2018) found that the volume of the right cerebellar crus was decreased in PTSD. Apart from that, Cheng et al. (2015), in a study with a multi-neuropsychiatric sample, reported decreased left cerebellum GMV in PTSD compared to obsessive-compulsive disorder subjects. A twin-study compared GMV variations in the midline vermis in combat-exposure PTSD individuals relative to non-PTSD twins (Levitt et al., 2006). No differences in GMV were reported by the authors, although the vermis was not parcellated into gray or white matter. Likewise, a study of intimate partner violence-related PTSD did not find significant cerebellar GMV alterations (Fennema-Notestine et al., 2002).

#### Anxiety disorders

Increased cerebellum GMV has been found in the left (Talati et al., 2013) and posterior (Talati et al., 2015) cerebellum of SAD participants, compared to controls. Volumetric decreases were reported in the vermis and left cerebellum after 12-weeks of treatment with escitalopram (Cassimjee et al., 2010) whereas Talati et al. (2015) reported cerebellar findings in the opposite direction following 8-week paroxetine treatment. Other studies did not report significant GMV cerebellar changes in SAD in comparison to controls (Zhang et al., 2022) or patients with obsessive-compulsive disorder (Cheng et al., 2015). In PD, Asami et al. (2009) identified a decreased left vermal GMV and sex-dependent differences with a reduced GMV in the right vermis of PD females compared to males. Conversely, in SP, increased GMV in the vermis was found in a combined dental and snake phobia group compared to controls (Hilbert et al., 2015).

## Discussion

It has been increasingly recognized that the functions of cerebellum extend into emotions, including fear and anxiety. The cerebellum could work as a complementary region to the amygdala in emotional reactivity and modulation (Strata, 2015), and the amygdala-cerebellum reciprocal link has been shown to be aberrantly functioning in post-traumatic stress and anxiety disorders (Nicholson et al., 2015; Thome et al., 2017; Sandman et al., 2020; Du et al., 2021; Park et al., 2022). Hence, this mini-review evaluated functional (task activity, resting state connectivity) and structural (gray matter) findings on the cerebellum reported in MRI studies of patients with PTSD or anxiety disorders.

Results showed that PTSD was the single most studied disorder, targeted in 63% of the included studies. The cerebellum tends to show hyperactivation in the task-based fMRI studies. Among the cerebellar subregions, the vermis (Ke et al., 2015, 2016), lobule VI (Rabellino et al., 2016; Naegeli et al., 2018), and crus I (Rabellino et al., 2018; Yuan M. et al., 2018; Awasthi et al., 2020; Nicholson et al., 2020), emerge as key cerebellar distinctive structures that could be involved in the symptomatology or developmental course of PTSD. The vermis has been highlighted for its role in enhancing episodic memory of emotional stimuli (Fastenrath et al., 2022), its contribution to fear-related memories maintenance (Strata et al., 2011) and is considered to be the limbic cerebellum (Klein et al., 2016).

It is noteworthy that the baseline vermis and left cerebellum hyperactivation in PTSD decreased over a long-term perspective, with baseline vermis activity being predictive of symptom improvement (Ke et al., 2016). It could be expected that successful treatment would contribute to downregulation of emotion-related cerebellar/vermal activity, although treatment effects should be further explored, especially considering the divergent findings noted for hydrocortisone interventions aimed at targeting the glucocorticoid system (Douglas et al., 2019; Metz et al., 2019; Schwab et al., 2020).

Across the anxiety disorders, results were mixed with regard to cerebellar hyper- or hypoactivation in task-based fMRI trials. Altered amygdala to cerebellum FC was noted in GAD (Park et al., 2022) and also following psychological intervention in SAD (Sandman et al., 2020). In SP, even when considering different types like blood and spider phobia, fMRI results seemed more homogeneous, showing increased activation of the left cerebellum and lobule VI. The cerebellar lobule VI is thought to be decisive for higher-order cognitive functions, such as working memory (Lange et al., 2015; Ashida et al., 2019). Studies supporting a functional topographic organization of the human cerebellum have noted idiosyncratic subregions to modulate sensorimotor, cognitive, and limbic processes (Stoodley and Schmahmann, 2010; Habas and Manto, 2018) with possible left-right lateralization (Baillieux et al., 2010). Following this, cerebellar activation patterns would depend largely on type of task or context (Schmahmann et al., 2019) which varied notably in the fMRI studies evaluated here.

With regard to resting-state fMRI findings, PTSD is marked by reduced FC between the cerebellum and the central executive network (CEN) (Vuper et al., 2021), in which the cerebellar crus II takes part. Disrupted CEN might reflect difficulty in concentration in adults with PTSD, and it has previously been shown to be affected in GAD (Kolesar et al., 2019). Altered crus I, that participates in the default mode network (DMN) (Halko et al., 2014), was found by Yin et al. (2011) measured with amplitude of low-frequency fluctuations. The DMN, also composed by cerebellar lobule IX, is further involved in mental imagery and long-term episodic memory processes (Habas et al., 2009), that are potential clinical features of PTSD (Zlomuzica et al., 2018; Petzold and Bunzeck, 2022; Almeida et al., 2023). Disrupted FC between the cerebellum and the dorsal anterior cingulate cortex (Chen et al., 2019) may reflect altered motor and cognitive processing involved in reward (Bush et al., 2002), while a reduced FC between the right vermis and the periaqueductal gray, bilateral culmen and left lingual might reflect a disrupted limbic system. The culmen, congregated in the anterior vermis, might be functionally connected to limbic structures, such as amygdala (Nicholson et al., 2015), hippocampus, nucleus accumbens and orbitofrontal cortex (Lange et al., 2015). Functional hypoconnectivity in anxiety disorders may be characterized by the interruption between the cerebellar nodes involved in the processing of social and aversive stimuli, such as Crus I (Chen et al., 2022), frontal (Yuan et al., 2017) and corticostriatal regions (Zhang et al., 2022). Interestingly, the cerebellar Crus I, shown to be altered in some studies on PTSD and anxiety disorders included herein (e.g., Yin et al., 2011; Yuan et al., 2017, Yuan M. et al., 2018; Rabellino et al., 2018; Nicholson et al., 2020) participates in hippocampus-dependent spatial navigation (e.g., Rondi-Reig et al., 2022) which may be impaired in PTSD (e.g., Smith et al., 2015). Still, further neuroimaging studies are needed to achieve better understanding of potentially altered cerebellum-hippocampus interactions (Watson et al., 2019) in PTSD and anxiety disorders.

Varying structural alterations in the cerebellum have been reported in PTSD (Blithikioti et al., 2022). Baldaçara et al. (2011, 2012) hypothesized that cerebellar hyperactivity might be characterizing the first post-trauma months, and as a consequence of this, cerebellar volume reduction may appear later. Likewise, MRI studies on cerebellar gray matter alterations in anxiety disorders do not provide a coherent picture since increases, decreases as well as null results have been reported even in circumscribed subregions like the vermis (Asami et al., 2009; Cheng et al., 2015; Hilbert et al., 2015). Also, the effects of antidepressant pharmacotherapy on cerebellar GMV have varied in direction (Cassimjee et al., 2010; Talati et al., 2015) which could be related to differences in antidepressant type, treatment duration, MR scanner and absence of control group in one of the studies. Further research aiming at finding sex differences at the cerebellar level could be relevant as sex-dependent results in PD were demonstrated (Asami et al., 2009).

Several limitations of the present mini-review should be considered. Firstly, the included studies differ widely in disorder type, characteristics of the samples, experimental design, intervention, neuroimaging modality, and type of analyses, which could limit the comparability. Small samples in several studies constrain the statistical power (Cremers et al., 2017) and reproducibility of findings (Turner et al., 2018). Moreover, in comparison to regions like the amygdala, only a small fraction of imaging studies has had an explicit cerebellar focus and, anatomically, results are frequently described in broad terms like anterior/posterior or right/left cerebellum, instead of providing a more specific localization (Strata, 2015). Also, the whole cerebellum has not been eligible for analysis in many trials (Fastenrath et al., 2022) as it has been common to remove part of the cerebellum from the field of view of the MR scanner (Anteraper et al., 2022).

In conclusion, this mini-review described and briefly evaluated functional and structural neuroimaging studies reporting on the cerebellum in adult participants with anxiety disorders or PTSD. While the vermis, acting as a limbic node in the cerebellum for emotions, could have a more prominent role in these disorders, it is also evident that the MRI studies evaluated herein report anatomically distributed findings, involving motor as well as non-motor regions of the cerebellum. The functionality of each cerebellar subregion is complex and the consistency and direction of cerebellar involvement across the disorders need further evaluation. To contribute to this, longitudinal and cross-sectional studies and large-scale networks, in combination with the use of ultra-high field MR scans offering improved anatomical precision, could provide a better understanding of the role of the cerebellum in post-traumatic stress and anxiety disorders.

## Author contributions

Both authors contributed to the design and conceptualization, literature searching and writing, and approved the final submitted version.

## Footnotes

1
https://pubmed.ncbi.nlm.nih.gov/
